# And Who Is My Neighbour?

**Published:** 1887-05-21

**Authors:** 


### "AND WHO IS MY NEIGHBOUR?"

Last week a thrill of horror was sent through the country by the publication of an account of a major in the Bulgarian army who was buried alive. This soldier of Eastern Europe, when returning to consciousness, seems to have succeeded in partially forcing the lid of his coffin, and to have endeavoured to sustain life by eating his fingers. The horrors of such an awakening are indescribable, but even they were forced to the background by the sense of hunger experienced by this officer when the first shock had passed away. It once happened to the present writer to find a case of genuine starvation in a tenement-house within a stone's-throw of Charing Cross. Those who have never seen such a case will not be able to realise what it is to starve to death by slow degrees. It may be well, therefore, to briefly describe the process.

Where a person suffers from an entire deficiency, or from an insufficient supply of food, death does not ensue, as a rule, until two-fifths of the whole weight of the body have been burnt off, that is, until the average weight of the sufferer has been diminished to the extent of forty per cent. The poor woman near Charing Cross presented all the characteristic symptoms. She was suffering violent pain, her countenance was pale and cadaverous, the eyes were wild and glistening, the breath hot, the mouth parched, and an intolerable thirst consumed the sufferer with unendurable torments. Her body was terribly emaciated, her skin covered with a brownish, dirty-looking, and offensive secretion, and her bodily strength was diminished to such an extent that, when she rose on our entering the room, she tottered like a drunken man. But worse, far worse than this, although the poor woman's voice was weak and whining, there was no delirium or mania, but, free from imbecility and without loss of reason, the agonising torments had to be endured in solitude and without hope.

The case here alluded to was that of an industrious and respectable woman who came to London as many hundreds, nay thousands, of others come, in the belief that there will be found an abundance of work and endless opportunity of making a good living. Her husband and one child first fell in the struggle for existence, which those who are acquainted with the poorer districts of London can alone realise and shudder at. Day by day there was the ceaseless, wearisome, disappointing search for work, which could seldom or never be found. Week by week the few things brought from the country home were pawned for food. Disheartened by failure, weakened by want, and depressed by the loss of her husband and child, this poor woman crept to the most miserable, because the cheapest, room she could find, and there, husbanding her resources to pay her rent, prepared herself for death by starvation. And why did she not seek shelter in the workhouse? Because, poor thing, shame—false shame, if you will—forbade such a step, and she had been taught it was better to be pinched, or half-starved, or even to "clam," than to go to the Union. Indeed, we had the greatest difficulty to secure her removal on a magistrate's order, as she resisted and resented our interference, preferring to starve alone in the miserable apartment where death had almost overtaken her when she was discovered.

Horrible, says the reader, and truly; but this woman was only one of scores in a similar predicament scattered amongst the vast population of London to-day. It appears from a return recently presented by the Home Office to the House of Commons that during the last year forty persons died in the metropolitan district upon whom the coroner's jury returned a verdict that their deaths were either caused by starvation, or were accelerated by privation and want. Alas, to its shame be it said, the county of Middlesex is responsible for four out of every five of these forty deaths, and no less than fifteen occurred within the area of the central or City division of London. Fifteen persons starved to death in the city of London, the centre of the money-market of the world! Truly a humiliating fact for Queen and people alike in this year of Jubilee. In East London there were sixteen deaths from starvation, whilst one person died of hunger in the west district, where wealth is so considerable and so common that a year ago the vicar of a West-end parish returned the sums sent to him from the Mansion House for his poor, on the ground that he knew of none in his district! Two were starved to death within an area of half a mile from the House of Commons, and three were found dead in the street in East London; and sadder still, in the Greenwich division of Kent the case of a dock labourer is recorded where the verdict returned was "slow starvation." But even this case was not the worst, for Richard Pratt was refused admission to the workhouse in the central or City division, and died in the street from the effects of severe weather and insufficient food. Such a record is shocking, when we realise that it concerns the largest, wealthiest, and most famous city, not only of the empire, but of the civilised world. And yet, alas, the forty deaths from starvation here recorded are but a fraction of the whole. Who shall number the many who fade out of existence for want of food and care in the metropolis alone, upon whom no inquest is held, and who are supposed to die from natural causes?

Can nothing be done to prevent these deaths from starvation? Truly much can be accomplished if the individual citizen will shake off his lethargy, and take prompt means to assert his individual interest and determination to prevent similar horrors from disgracing his day, his city, and his nation. Those who get their living in the city of London, as they return in the evening cannot fail to be struck by sights in the streets where the poor and starving appeal to their sympathy and support. We are not prepared to advise, nay, we would rather censure, the easy-going benevolence of the passer-by who gives a small dole, and goes on his way feeling that he is better than his neighbour. These doles place a premium upon the importunities of the indolent and mendacious, to the loss of the really suffering poor. Rather would we urge every man and every woman to determine, either by communication with the committee of the Mendicity Society within his district, or by the more self-denying course of personal examination and inquiry, to follow up each case that comes under his notice as he returns home in the evening. If everybody, or even a sensible proportion of the better-to-do classes, were to determine to follow up all the cases they met with in the streets which they pass through on their way home each evening, then much would be done to clear the streets of the undeserving, and to secure adequate sustenance for the starving poor.

The churches of all denominations do much, bnt they are crippled for want of assistance in their efforts to keep themselves informed of the circumstances of the new residents who come into their districts, and who need counsel and help in various ways. It is not for us to do more than suggest a course of action, and to enforce a lesson from the cruel facts here recorded; but if each man and woman will ask themselves the question, "And who is my neighbour?" and will ponder and dwell upon the terrible truths and the awful agonies of the forty starved to death in London last year, something will be done to relieve our day and generation from a reflection so burdensome as to be well-nigh insupportable.

